# Dataset for Vaginal Human Papillomavirus infection among adolescent and early adult girls in Jos, Nigeria

**DOI:** 10.21203/rs.3.rs-2763660/v1

**Published:** 2023-04-04

**Authors:** Nanma T. Cosmas, Lohya Nimzing, Daniel Z. Egah, Ayotunde O. Famooto, Sally N. Adebamowo, Clement A. Adebamowo

**Affiliations:** University of Jos; University of Jos; University of Jos; University of Maryland School of Medicine, Baltimore, Maryland.; University of Maryland School of Medicine, Baltimore, Maryland.; University of Maryland School of Medicine, Baltimore, Maryland.

**Keywords:** HPV infection, sexual behaviours, HPV vaccine knowledge, HPV types, Adolescent and early adult girls

## Abstract

**Objectives:**

To assess risk factors for HPV infection, determine knowledge about HPV vaccines, assess willingness to receive the HPV vaccine among adolescent and early adult girls in Nigeria, we administered a structured questionnaire. We also collected samples to determine the prevalence and patterns of HPV infections.

**Data description:**

The dataset contains the responses of 205 participants from 10 randomly selected public and private secondary schools in Jos, Nigeria. The data includes information on risk factors for HPV infections such as sexual behaviours, knowledge about HPV vaccine and willingness to receive the vaccine. This is valuable information that can be compared to data from studies in other environments or to determine changes in the pattern of risk factors and HPV prevalence in this population over time.

## Background

Persistent high risk Human papillomavirus (HPV) infection is a necessary cause of cervical cancer [1]. HPV infections are very common and usually transient. The first episode typically occurs shortly after sexual debut in adolescence and early adulthood [2,3]. Several factors contribute to the risk of HPV infections. These include risky sexual behaviours, sexual hygiene, and substance abuse [4,5][6]. Although vaccines are available for the prevention of HPV infection, these are not yet readily or widely available in Nigeria. Hence the need to establish current prevalence, pattern, and risk factors for HPV infection young girls in this population.

We collected data from 205 adolescent and early adult girls. Details of the enrolment process in the study has been published elsewhere [7]. After enrolment, and obtaining informed consent and assent, research nurses from the Jos University Teaching Hospital (Nigeria) interviewed each participant in private using a structured questionnaire. Young nurses were used to encourage the girls to relax and be more open to participating in this study, particularly in discussions related to sexual matters. The study questionnaire included questions on socioeconomic and family characteristics, sexual preferences, sexual debut, condom use, sexual behaviour including masturbation, menstrual, and personal hygiene practices.

We also collected data on knowledge related to HPV infections and HPV vaccine, and willingness to receive the vaccine. The questionnaire design was guided by the research questions and literature review [8–10]. We pretested the questionnaire in 10 people to check for correctness and ease of administration. The participants were trained to self-collect vaginal samples which were tested for HPV infection using DEIA/LIPA_25_.

This dataset will contribute immensely to comparisons of HPV infection among other population in Nigeria and other countries. Researchers involved in data mining and analysis of secondary data will also find the dataset useful.

## Data Description

The dataset (**data file 1 on** Table 1) [11] contains responses to 205 questionnaires from adolescent and early adult girls. Because some of the girls were minors and unable to give consent in their own cognizance, we obtained consent and assent as required [7]. The girls were enrolled from 10 randomly selected schools in Jos, Nigeria after meeting the inclusion criteria of being within the age range and haven provided informed consent and/or ascent. Some 50.7% (104/205) of the participants were recruited from public schools while 49.3% (101/205) were from private schools. The mean age (SD) of the girls was 14.7 (1.97) years with a minimum of 9 and maximum age of 20 years old. Most of the girls (145/205, 70.7%) had attained menarche and the earliest age at menarche was 9 years. Instead of socio-economic status, we used principal component analysis (PCA) to compute wealth index using data on ownership of household items and availability of household facilities such as type of house they live in, type of toilet facilities, main source of cooking fuel etc [12].

To assess the sexual risk factors for HPV infections, we collected data on the sexual history of the girls. Only 9.3% (19/186) of the girls reported history of penetrative vaginal sexual intercourse. Most of the girls (57.9%, 11/19) who reported sexual intercourse first experienced it at 15 years of age or less and 15.8% (3/19) experienced it before the age of 10 years. We also collected information on the participants’ sexual partners. Vaginal douching practices were reported by 16.6% of the girls and most of the girls who practice douching gave ‘It is part of taking a bath’ as the main reason why they engaged in the practice. Most of the girls (89.0%) took their bath at least twice daily when menstruating and 61.4% regularly bathe twice daily. Participants also shared personal clothes (24.9%) and pants (5.4%). Some girls reported history of masturbation, use of toys for masturbation, use of condoms and knowledge about ways to prevent pregnancy.

Of the 205 participants, only 5 (2.4%) had heard about HPV vaccine and only 2 (1.0%) had received the vaccine. Some 32.4% (66/205) girls had no interest in receiving HPV vaccination and the reason for their unwillingness was cost of the vaccine. Almost all the girls (196, 95.6%) would receive the vaccine if it is free or subsidized.

The prevalence of any HPV infections among the participants was 13.2% (27/205) and 15 different HPV types were detected with HPV type 52 being the commonest. Participants had single or multiple HPV infections, with 5 HPV types being the highest number of types of HPV found in multiple infections.

## Limitation

Most participants reported that they had never had any sexual contact. Discomfort about reporting sexual history among participants in this study of young and early adult girls may cause information bias [13]. Our small sample size is small and may impact the assessment of risk factors of HPV infection and characterization of the pattern of HPV types. Participants were enrolled from private and public schools so the results may be different if unschooled adolescent and early adult girls are included in the study.

## Figures and Tables

**Table 1:** Overview of data set

Label	Name of data file/data set	File types (file extension)	Data repository and identifier (DOI or accession number)
Data file 1	HPV among adolescent and young adult grils_dataset.xlsx	Excel file (.xlsx)	Figshare (https://doi.org/10.6084/m9.figshare.20334534.v1)

## Data Availability

The data described in this Data note can be freely and openly accessed on https://doi.org/10.6084/m9.figshare.20334534.v1.
